# Dynamic Interactions between Emotion Perception and Action Preparation for Reacting to Social Threat: A Combined cTBS-fMRI Study

**DOI:** 10.1523/ENEURO.0408-17.2018

**Published:** 2018-07-02

**Authors:** Tahnée Engelen, Minye Zhan, Alexander T. Sack, Beatrice de Gelder

**Affiliations:** Department of Cognitive Neuroscience, Faculty of Psychology and Neuroscience, Maastricht University, Maastricht, 6229 EV, The Netherlands

**Keywords:** amygdala, brain stimulation, emotion, emotion body processing, parietal cortex, premotor cortex

## Abstract

Expressions of emotion are powerful triggers for situation-appropriate responses by the observer. Of particular interest regarding the preparation of such adaptive actions are parietal and premotor cortices, given their potential for interaction with the amygdala (AMG), which is known to play a crucial role in the processing of affective information and in motor response. We set out to disentangle the respective roles of the inferior parietal lobule (IPL) and ventral premotor cortex (PMv) in humans in the processing of emotional body expressions by assessing remote effects of continuous theta burst stimulation (cTBS) in the action network and in AMG. Participants were presented with blocks of short videos showing either angry or neutral whole-body actions. The experiment consisted of three fMRI sessions: two sessions were preceded by stimulation of either right IPL (rIPL) or right PMv (rPMv); and a third session assessed baseline activity. Interestingly, whereas at baseline the left AMG did not differentiate between neutral and angry body postures, a significant difference between these conditions emerged after stimulation of either rIPL or rPMv, with much larger responses to angry than to neutral stimuli. In addition, the effects of cTBS stimulation and emotion were also observed in two other action-relevant areas, the supplementary motor area and the superior parietal cortex. Together, these results show how areas involved in action and emotion perception and in action preparation interact dynamically.

## Significance Statement

When confronted with social threat, the brain must prepare situation-adaptive responses. Previous studies have shown that inferior parietal lobule (IPL) and ventral premotor cortex (PMv) are critically involved in social threat perception and that amygdala is triggered by emotional valence of the stimuli. So far, the causal relation among IPL, PMv, and amygdala is not clear. By combining continuous theta burst stimulation with fMRI, we show that the stimulation of IPL and PMv induces significant differences between the perception of angry versus neutral bodies in amygdala. Furthermore, stimulation of these areas led to significant interaction effects between the emotion presented and the site stimulated in a number of remote parietal and motor areas. Our findings demonstrate the involvement of IPL and PMv in emotion body processing, and interaction between these areas and with the amygdala during this process.

## Introduction

Emotional expressions do not just communicate affective information to the observer, but more importantly they are powerful triggers for adapting one’s behavior. To illustrate, one expects that a threatening body expression prompts a defensive reaction in the observer. While many studies have investigated this process from the perception side, fewer have looked at the reactive aspects. Many cortical and subcortical regions play a prominent role in as much as they are part of information-processing networks, and foremost among them seems to be the amygdalae (AMGs). What is still largely unknown is how these multiple network components interact to trigger the well known behavioral effects of perceiving emotional signals like, for example, the sight of an aggressive posture. Previous studies have shown that readying the brain for dealing with emotional signals involves visual processes, but the fact that this is also associated with changes in activity in motor structures is less understood (de Gelder et al., 2004; Borgomaneri et al., 2015a,b). The goal of the present study was to clarify the latter issues.

The inferior parietal lobule (IPL) is ideally located to play the role of a hub in which emotion perception is transitioned into an action response. IPL receives input from the visual system (Caspers et al., 2011) and has connections to premotor cortex (PM; Makris et al., 2005; Hoshi and Tanji, 2007; Mars et al., 2011), where actions are prepared. Presumably, given this role, IPL differentiates between neutral and emotional actions and may be involved in fast responses to the latter. Indeed, evidence for this was provided using MEG. Meeren et al. (2016) showed a response in right posterior parietal cortex to fearful, compared with neutral body postures as early as 80 ms after stimulus onset. A causal involvement of IPL in emotion body processing was first demonstrated by applying on-line transcranial magnetic stimulation (TMS) during a delayed match-to-sample task. Results showed an enhancement in performance in a fearful body condition (Engelen et al., 2015), providing evidence for an increased sensitivity to emotional body stimuli under conditions of IPL stimulation.

PM is involved in action preparation and execution (Picard and Strick, 2001; Hoshi and Tanji, 2007), with the ventral subpart of PM (PMv) specifically involved in space perception and the understanding of actions (Rizzolatti et al., 2002; Urgesi et al., 2007a,b). Several neuroimaging experiments have found emotion-specific activation of PMv (Pichon et al., 2008; Kret et al., 2011; Calbi et al., 2017). A TMS study targeting PM found an increase in reaction times and false alarms, specifically in response to faces expressing fear or anger (Balconi and Bortolotti, 2013).

In view of the findings that emotional signals trigger activity in IPL and PM, a following question concerns the further details of the mechanism whereby these areas are influenced by affective information of the stimulus. The AMG is viewed as a major player in processing stimulus valence in humans (Davis and Whalen, 2001; Baxter and Murray, 2002; Salzman and Fusi, 2010) and has been placed in the center of regulating defensive motor responses (LeDoux and Daw, 2018). Amygdala activation is also associated with viewing whole-body expressions of threat (Hadjikhani and de Gelder, 2003; Poyo Solanas et al., 2018), but this varies with the type of task and the relative roles of attention and awareness (de Gelder et al., 2012). Combining findings about the activation of motor structures and the role of the amygdalae indicates that an important part of the underlying mechanism involves interactions between the amygdalae and motor structures. This is also suggested by a recent study using diffusion-weighted imaging and probabilistic tractography to investigate connectivity between AMGs and sensorimotor regions (Grèzes et al., 2014).

Findings about the involvement of premotor and parietal regions as well as the amygdalae in the perception of whole-body expressions of emotion are consistent with a network perspective on emotion perception (Dubois et al., 2017; Pessoa, 2017), and the notion that emotion processing is implemented in brain networks that dynamically interact in situations of threat. In this vein, the present study tested directly the existence of an emotion-dependent dynamic interaction between action-related areas and AMG. We used an off-line combined TMS–functional magnetic resonance imaging (fMRI) design to specifically manipulate neural activity within IPL and PMv using continuous theta burst stimulation (cTBS) and assessed network interaction effects within this motor–emotion circuitry during the passive viewing of either neutral or angry body emotions. During three fMRI sessions, participants received either off-line stimulation over IPL or PMv, or no stimulation (baseline fMRI). During the acquisition of fMRI data, immediately following stimulation participants were presented with dynamic stimuli depicting either angry or neutral whole-body actions. We expected to find an interaction effect between the site of stimulation and the valence of the presented stimulus, demonstrating (1) the emotion-specific interplay between amygdala and action-related areas of the brain to differentiate between the valence of the stimuli and (2) a valence-specific mediating role of parietal and motor areas on amygdala response patterns. Additionally, we explored whether any condition-specific effects of stimulation could be observed in two areas that are well established as being important in the perception of bodies and biological motion, namely extrastriate body area (EBA) and posterior superior temporal sulcus (pSTS). Although these areas are reported in many studies on emotion bodies, including when the same stimuli as in the current experiment are used (Kret et al., 2011), the specific involvement of these areas in the recognition of affective meaning of body images is still unclear.

## Materials and Methods

### Participants

Seventeen healthy participants completed all three sessions of the experiment (5 males; mean age, 23 years; SD, 2.2). Fifteen participants were right handed, and all had normal or corrected-to-normal vision. Participants signed a written consent form before participating and were rewarded for their participation in vouchers. Each participant was screened for fMRI and TMS safety, and none had a history of neurologic disorders. Given the demanding design of the experiment (immediately entering the scanner after receiving cTBS), only participants with prior TMS experience were recruited. Additionally, to have a counterbalancing order of the sessions, a pre-existing T1 image was necessary for localizing the stimulation sites, and therefore we recruited participants who had previously participated in other fMRI experiments. The study was approved by the local ethics committee and was performed in accordance with the Declaration of Helsinki.

### Stimuli

The same stimulus dataset was used as reported in the study by Kret et al. (2011). Stimuli consisted of short 1 s video clips, each showing a male actor performing either angry or neutral whole-body movements recorded against a homogeneous background. For the neutral movement, the actor raised a hand in front of the face while coughing, and in the angry videos the actor raised a fist in front of the trunk. Eleven different clips were performed by six different actors. The clips were presented without sound, and the stimulus size was 720 × 576 pixels.

### TMS stimulation and site localization

Before the start of two of the fMRI session, cTBS (three pulses at 50 Hz, every 200 ms for a total of 600 pulses; Huang et al., 2005) was applied at 80% of the active motor threshold [mean intensity, 25 maximum stimulator output (MSO); SD, 3.5 MSO] using an MC-B70 figure-of-eight coil and Magpro X100 stimulator (Medtronic Functional Diagnostics A/S). Either right PMv (rPMv) or right IPL (rIPL) were targeted based on individual macroanatomic landmarks.

**Figure 1.**
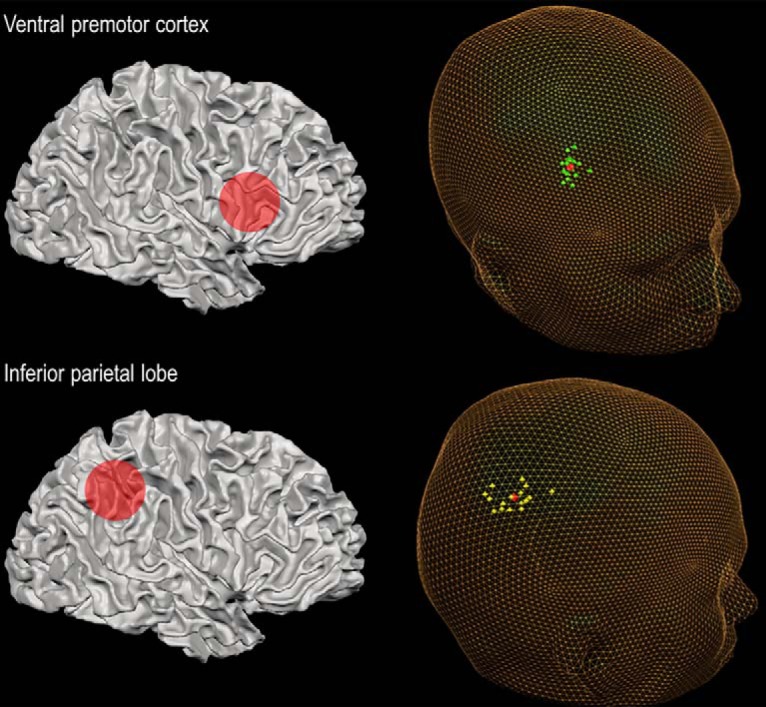
The two stimulation sites for cTBS consisted of the right ventral premotor cortex and right inferior parietal lobule. Both target points were localized using individual brain anatomy (for details, see TMS stimulation and site localization), and cTBS stimulation was guided by neuronavigation. Individual target points are displayed in Talairach space for rPMv (green) and rIPL (yellow). The red spheres indicate the average stimulation coordinates.

The rIPL was located by identifying a point that lies directly posterior to the intraparietal sulcus (IPS) at the caudal end of the posterior branch of superior temporal sulcus (the same approach as used in the study by Engelen et al., 2015). IPL is a large and heterogeneous area consisting of seven separate cytoarchitectonic sections, two of which are located in the angular gyrus (suggestive of functional segregation; Caspers et al., 2006; Igelström and Graziano, 2017). These subdivisions demonstrate large interindividual variability and do not always correspond to macroanatomic landmarks, which we used in our localization approach. Therefore, we cannot exclude that different subsections of IPL were targeted for different participants. For most participants, the stimulated area likely corresponds to the location of the angular gyrus. For rPMv, we selected a point directly below the intersection of inferior frontal sulcus and precentral gyrus, an approach similar to that used by Cattaneo et al. (2010) and Tremblay et al. (2012). See Table 1 for the average Talairach coordinates per stimulation site, and Figure 1 for a representation of individual stimulation sites in Talairach space. For both stimulation sites, the coil was positioned with the handle pointing backward and outward at a 45˚angle from the midsagittal axis.

**Table 1.** Average Talairach coordinates per stimulation site ± SD

Stimulation site	*x*	*y*	*z*
Inferior parietal lobule	41 ± 7	−62 ± 9	43 ± 5
Ventral premotor cortex	50 ± 6	11 ± 4	29 ± 5

Values are reported as the mean ± SD.

### Procedure

The experiment consisted of three sessions, performed on separate days with at least 1 week in between each session. The order of sessions was counterbalanced as much as possible; however, with anatomic data necessary to perform the neuronavigation not available for some participants, most participants (*N* = 9) started with a baseline session (the other participants started with either IPL or PMv stimulation). During two of the sessions cTBS was applied over either rIPL or rPMv. During these TMS sessions, the motor threshold was established by moving the coil over primary motor cortex (M1) until an optimal position was found for eliciting muscle twitches in the hand muscles. After this, the stimulation intensity was decreased until a threshold was found at which 5 of 10 pulses still evoked a motor response while there was some tension in the hand muscles. Next, the correct coil location on the scalp was determined for each stimulation site by using the BrainVoyager TMS Neuronavigator software (Brain Innovation). Once the coil was in the correct position, the cTBS protocol was applied, after which participants were moved to the scanner environment as quickly as possible (the first functional run was started <10 min after stimulation). The participant was placed in the scanner, and two functional runs were acquired in which the stimuli portraying dynamic neutral or angry actions were presented. Each functional run lasted 8 min (16 min of task in total), so the total scanning time fell within the window of assumed effectivity for cTBS (cTBS effects are reported to last up to 60 min in the protocol for an application of 40 s; Huang et al., 2005). Participants were instructed to pay attention to the presented stimuli throughout the functional run. Given the short amount of time that attention had to remain focused on the stimuli, none of the participants mentioned issues with managing this. However, there was no formal check of attention such as catch trials, which has to be noted as a limitation of the study. It also has to be noted that the order of angry and neutral blocks was not randomized within a run. The session in which no stimulation was applied followed the same scanning procedure, with the addition of two functional localizers at the end of the scan (for details, see Functional localizers).

### Scanning parameters

Functional images were acquired using a 3 T MAGNETOM Prisma fit scanner (Siemens) with a 64-channel head-neck coil. A gradient-echo EPI sequence was used [repetition time (TR) = 2 s; echo time = 31 ms; voxel size = 2 × 2 × 2 mm^3^; 64 slices; no gaps; multiband acceleration factor = 2; flip angle = 77°], providing whole-brain coverage. A high-resolution T1-weighted MPRAGE anatomic scan was acquired for each participant during the baseline session (voxel size = 1 × 1 × 1 mm; 192 slices).

The main experiment started with a fixation period of 6 TRs, followed by blocks of stimuli of 6 TRs separated by fixation periods of 6 TRs, and ended with a fixation period of 12 TRs. During each block, a total of 12 dynamic whole-body stimuli was shown, depicting either angry or neutral movements. The main task contained two functional runs; during each run, 16 blocks of stimuli were presented. Per session, a total of 432 volumes were acquired for the main task.

### Functional localizers

To identify individual ROIs of EBA, the same functional localizer was used as described in the study by Engelen et al. (2015). Static images of five different categories of images (bodies, faces, houses, tools, and words) were shown pseudorandomized in a block design, with seven blocks per condition. Each block lasted six TRs interspersed with fixation periods of six TRs. The same scanning parameters as in the main task were used. A total of 432 volumes were acquired for this localizer. To identify bilateral EBA, a contrast was made between the responses to bodies and houses and the cluster selected that showed the greatest relative activation for the condition of the bodies (in case more than one cluster was observed in the same hemisphere, the clusters were merged into one).

In order to determine a functional ROI in right pSTS (rpSTS), another localizer was used in which short dynamic clips of male faces and bodies were presented in a block design. Each block consisted of eight 1 s dynamic stimuli, lasting a total of 4 TRs. These blocks were interspersed with fixation periods of 4 TRs. In total, 10 blocks of each stimulus condition were presented, and a total of 168 volumes were acquired for this localizer.

### Data preprocessing

Functional and anatomic brain-imaging data were preprocessed and analyzed off-line using BrainVoyager QX (Brain Innovation BV). Functional data were slice-scan time corrected, and 3D motion was corrected using sinc interpolation. To correct for low-frequency drifts in the data, high-pass temporal filtering was applied. Spatial smoothing with a Gaussian kernel of 6 mm FWHM was applied to the acquired images in the main task, and smoothing of 4 mm was applied to the functional localizers. All functional runs of the three separate sessions were aligned to the anatomic scan acquired during the baseline session. To enable analyses on the group level, individual data were spatially transformed into Talairach space. A brain mask was created by averaging the first volume of each functional run of all participants and removing the scalp. This mask was applied during the cluster threshold correction.

### GLM analysis

First, for each individual participant, a GLM map was created for each of the sessions using the predictors “angry bodies” and “neutral bodies.” The *z*-transformed 3D motion parameters were added as confound predictors in the model. The maps of all participants and sessions were combined as inputs for a random effects (RFX) ANOVA group analysis. This RFX ANOVA included the factors emotion (neutral and angry bodies) and session [reflecting the different stimulation conditions (cTBS over rIPL, cTBS over rPMv, or baseline)]. The resulting maps revealed by the ANOVA were corrected for multiple comparisons by cluster threshold estimation (α = 0.05; initial threshold set at *p* = 0.001; Monte Carlo simulation, *n* = 5000).

### ROI analysis

For each participant, bilateral amygdalae were segmented based on the anatomic data to isolate any effects of emotion and cTBS in the amygdalae. To assess the local effects of cTBS stimulation, for each participant an ROI for rIPL and rPMv was created. This was done by first creating a gray matter segmentation of the right hemisphere, and then placing a spherical ROI of 10 mm^3^, centered at the individual stimulation coordinates. For each of the segmented AMG clusters, the rIPL and rPMv ROIs, as well as the functionally determined ROIs for bilateral EBA and rpSTS, β values (percentage signal changes) were extracted from the functional runs for each emotion (neutral, angry) and stimulation condition (cTBS over rIPL, cTBS over rPMv, or baseline). These β values were then analyzed in a 2 × 3 repeated-measures ANOVA.

## Results

### Whole-brain random-effects ANOVA

#### Main effect of emotion

Clusters showing a main effect of emotion were observed in bilateral fusiform gyri, anterior inferior frontal gyrus, bilateral amygdala, right pulvinar, and superior parieto-occipital cortex (SPOC). For an overview of all clusters, see Table 2. For all clusters, the activation for angry bodies was stronger than that for neutral bodies, except for the SPOC cluster (cluster 5 in Table 2), for which there was a stronger activation for neutral than for angry bodies.

**Table 2.** Clusters found for the main effect of emotion (α = 0.05, cluster size corrected)

Anatomical description	Hemisphere	Cluster size	*x*	*y*	*z*	*F* value	*p* value
Widespread occipital/fusiform gyrus	R	16,826	43	−67	0	85.09	0.000000
Anterior inferior temporal gyrus	R	363	36	−5	−36	45.78	0.000005
Amygdala	R	347	25	−1	−12	56.63	0.000001
Pulvinar	R	354	17	−27	2	45.70	0.000005
Superior parieto-occipital cortex	R/L	2864	1	−59	0	54.17	0.000002
Widespread occipital/fusiform gyrus	L	22,222	−15	−89	−2	75.61	0.000000
Amygdala	L	697	−35	−1	−12	45.41	0.000005

Cluster size is reported in number of voxels (voxel size = 1 mm³). Reported values (coordinates, *F* value, and *p* value) reflect cluster peak. L, Left; R, right.

#### Main effect of session

Clusters showing a main effect of session (baseline, cTBS over rIPL, or cTBS over rPMv) were observed in right ventromedial prefrontal cortex (vmPFC), left posterior cingulate cortex (PCC), left precuneus, left superior parietal lobe (SPL), and left PMv (lPMv). The cluster in lPMv additionally showed an emotion × session interaction, details of which are reported in the section discussing the interaction findings.

**Figure 2.**
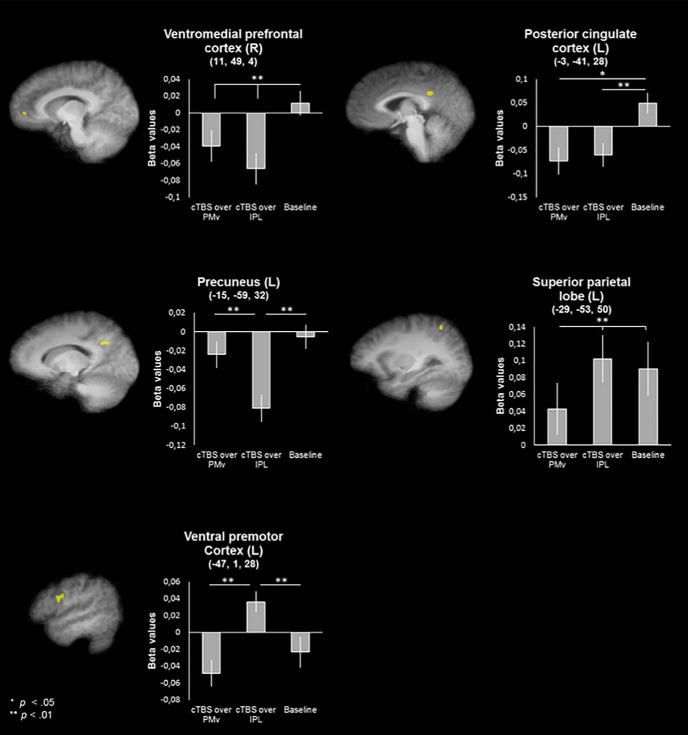
Clusters showing a significant main effect of session displayed on an averaged anatomy of all participants in Talairach space (*p* = 0.05, cluster size corrected). For details about cluster size and statistical significance, see Table 3.

Pairwise comparisons showed that the clusters in vmPFC and PCC showed lower activity after cTBS over both rPMv (*p* = 0.007 and *p* = 0.033, respectively, Sidak correction) and rIPL (*p* = 0.001 and *p* < 0.001) compared with baseline, whereas the cluster in precuneus showed lower activity after cTBS over rIPL compared with both baseline (*p* = 0.003) and cTBS over rPMV (*p* = 0.003). The cluster in SPL significantly decreased in activation after cTBS over rPMv compared with baseline (*p* = 0.002) and cTBS over rIPL (*p* = 0.002). Last, the cluster in lPMv showed an increase in activation after cTBS over rIPL compared with both other sessions (*p* = 0.002 and *p* = 0.008). For a detailed overview of all clusters found for the main effect of session, see Table 3 and Figure 2.

**Table 3.** Clusters found for the main effect of session (α = 0.05, cluster size corrected)

Anatomical description	Hemisphere	Cluster size	*x*	*y*	*z*	*F* value	*p* value
Ventromedial prefrontal cortex	R	112	11	49	4	15.37	0.000021
Posterior cingulate cortex	L	197	−3	−41	28	14.87	0.000027
Precuneus	L	108	−15	−59	32	11.97	0.000131
Superior parietal lobe	L	107	−29	−53	50	15.47	0.000020
Ventral premotor cortex	L	255	−47	1	28	11.73	0.000151

Cluster size is reported in number of voxels (voxel size = 1 mm³). reported values (coordinates, *F* value, and *p* value) reflect cluster peak. L, Left; R, right.

#### Emotion by session interaction effect

Clusters reflecting an emotion × session interaction effect were found in right lingual gyrus, bilateral SPL, left SPOC, cerebellum, precuneus, and lPMv.

**Figure 3.**
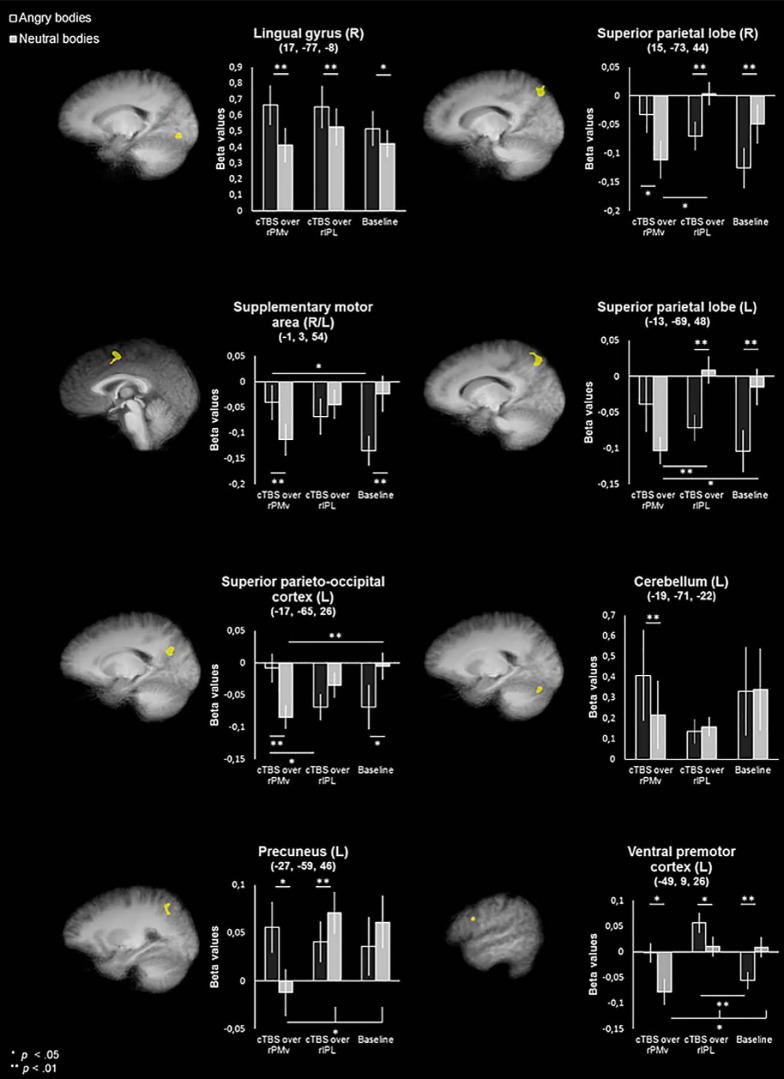
Clusters showing a significant emotion × session interaction displayed on an averaged anatomy of all participants in Talairach space (*p* = 0.05, cluster size corrected). For details about cluster size and statistical significance, see Table 4.

*Post hoc* tests were completed for each cluster to disentangle the significant differences underlying the interaction. For a visual overview of these significant differences, see Figure 3.

**Table 4.** Clusters found for the emotion × session interaction (α = 0.05, cluster size corrected)

Anatomical description	Hemisphere	Cluster size	*x*	*y*	*z*	*F* value	*p* value
Lingual gyrus	R	287	17	−77	−8	13.76	0.000049
Superior parietal lobe	R	1077	15	−73	44	17.98	0.000006
SMA	R/L	505	−1	3	54	12.77	0.000084
Superior parietal lobe	L	802	−13	−69	48	15.47	0.000020
Superior parieto-occipital cortex	L	340	−17	−65	26	14.99	0.000025
Cerebellum	L	164	−19	−71	−22	12.12	0.000120
Precuneus	L	354	−27	−59	46	14.52	0.000032
Ventral premotor cortex	L	113	−49	9	26	13.20	0.000066

Cluster size is reported in number of voxels (voxel size = 1 mm³). Reported values (coordinates, *F* value, and *p* value) reflect cluster peak. L, Left; R, right.

**Figure 4.**
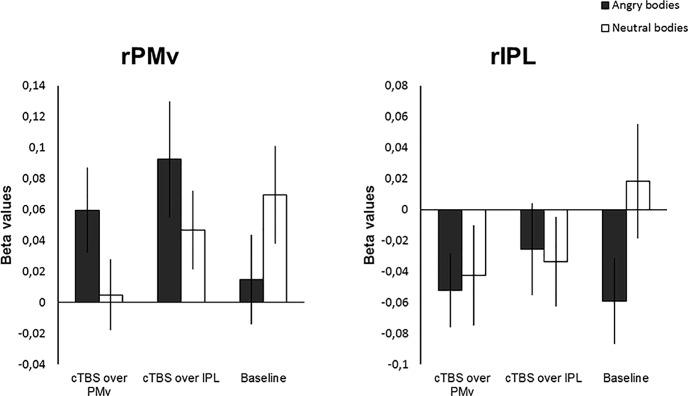
Results from ROI analysis based on individual stimulation sites (rPMv and rIPL). β values in rPMv showed a significant emotion × session interaction effect, whereas in rIPL no significant effects were found.

The cluster in the lingual gyrus showed a main effect for emotion in all three sessions (*F*_(1,16)_ = 44.153, *p* < 0.001, η^2^*_p_* = 0.734 in the PMv session; *F*_(1,16)_ = 9.460, *p* = 0.007, η^2^*_p_* = 0.372 in the IPL session; and *F*_(1,16)_ = 7.037, *p* = 0.017, η^2^*_p_* = 0.305, in the baseline session; Fig. 4). To further follow up on the interaction and see whether the difference between angry and neutral bodies became relatively larger between sessions, we calculated the difference between the angry condition of two sessions and subtracted from that the difference between the neutral conditions of the same sessions. *t* Tests (two tailed) showed that the difference between angry and neutral bodies is relatively larger when comparing PMv to baseline (*t*_(16)_ = 2.896, *p* = 0.011) and to IPL (*t*_(16)_ = 2.794, *p* = 0.013), but not when comparing IPL to baseline (*t*_(16)_ = 0.642, *p* = 0.530).

The cluster in right SPL showed a significant main effect of emotion in the PMv session (*F*_(1,16)_ = 5.567, *p* = 0.031, η^2^*_p_* = 0.258), the IPL session (*F*_(1,16)_ = 14.331, *p* = 0.002, η^2^*_p_* = 0.472), and the baseline session (*F*_(1,16)_ = 9.775, *p* = 0.007, η^2^*_p_* = 0.379). Within the angry condition, there was a main effect of session (*F*_(2,32)_ = 4.104, *p* = 0.026, η^2^*_p_* = 0.204), which was caused by differences between the PMv and baseline sessions (*p* = 0.015). In the neutral condition, there was also a main effect of session (*F*_(2,32)_ = 5.120, *p* = 0.012, η^2^*_p_* = 0.242), which was driven by a significant difference between the PMv and IPL sessions (*p* = 0.015).

The cluster in SMA showed a main effect of emotion in the PMv session (*F*_(1,16)_ = 15.652, *p* = 0.001, η^2^*_p_* = 0.494) and the baseline session (*F*_(1,16)_ = 10.038, *p* = 0.006, η^2^*_p_* = 0.385), but not in the IPL session (*F*_(1,16)_ = 0.685, *p* = 0.420, η^2^*_p_* = 0.041). Within the angry condition there was a main effect of session (*F*_(2,32)_ = 3.912, *p* = 0.030, η^2^*_p_* = 0.196), caused by differences between the PMv and baseline session (*p* = 0.040). In the neutral condition there was no main effect of session (*F*_(2,32)_ = 3.041, *p* = 0.062, η^2^*_p_* = 0.160).

The cluster in left SPL (lSPL) showed a main effect of emotion within the IPL session (*F*_(1,16)_ = 11.441, *p* = 0.004, η^2^*_p_* = 0.417) and baseline session (*F*_(1,16)_, 10.824, *p* = 0.005, η^2^*_p_* = 0.404), but not in the PMv session (*F*_(1,16)_ = 3.825, *p* = 0.068, η^2^*_p_* = 0.193). Within the angry condition, there was no main effect of session (*F*_(2,32)_ = 1.794, *p* = 0.183, η^2^*_p_* = 0.101). Within the neutral body condition, however, there was a main effect of session (*F*_(2,32)_ = 10.256, *p* < 0.001, η^2^*_p_* = 0.391), which was driven by significant differences between the PMv and IPL sessions (*p* < 0.001), and the PMv and baseline sessions (*p* = 0.015).

The cluster in SPOC showed a main effect of emotion in the PMv session (*F*_(1,16)_ = 17.390, *p* = 0.001, η^2^*_p_* = 0. 521) and the baseline session (*F*_(1,16)_ = 5.780, *p* = 0.029, η^2^*_p_* = 0.265), but not in the IPL session (*F*_(1,16)_ = 3.529, *p* = 0.079, η^2^*_p_* = 0.181). Within the angry condition, there was a main effect of session (*F*_(2,32)_ = 3.664, *p* = 0.037, η^2^*_p_* = 0.186), which was driven by a significant difference between the PMv and IPL sessions (*p* = 0.019). Within the neutral condition, there was a main effect of session (*F*_(2,32)_ = 4.336, *p* = 0.022, η^2^*_p_* = 0.213), which was driven by differences between the PMv and baseline sessions (*p* = 0.007).

The cluster in cerebellum showed only a main effect of emotion in the PMv session (*F*_(1,16)_ = 8.885, *p* = 0.009, η^2^*_p_* = 0.357), but not in the IPL session (*F*_(1,16)_ = 0.433, *p* = 0.520, η^2^*_p_* = 0.026) or the baseline session (*F*_(1,16)_ = 0.095, *p* = 0.761, η^2^*_p_* = 0.006). There was no main effect of session in either the angry condition (*F*_(2,32)_ = 1.110, *p* = 0.342, η^2^*_p_* = 0.065) or the neutral condition (*F*_(2,32)_ = 0.648, *p* = 0.530, η^2^*_p_* = 0.039).

The cluster in precuneus showed a main effect of emotion in both the PMv session (*F*_(1,16)_ = 9.309, *p* = 0.008, η^2^*_p_* = 0.368) and the IPL session (*F*_(1,16)_ = 10.222, *p* = 0.006, η^2^*_p_* = 0.390), but not in the baseline session (*F*_(1,16)_ = 1.673, *p* = 0.214, η^2^*_p_* = 0.095). Within the angry condition, there was no main effect of session (*F*_(2,32)_ = 0.456, *p* = 0.638, η^2^*_p_* = 0.028). Within the neutral condition, there was, however, a main effect of session (*F*_(2,32)_ = 7.416, *p* = 0.002, η^2^*_p_* = 0.317), which was driven by differences between the PMv and IPL sessions (*p* = 0.016), as well as differences between the PMv and baseline sessions (*p* = 0.016).

The cluster in lPMv showed a main effect of emotion in each of the sessions (PMv: *F*_(1,16)_ = 7.238, *p* = 0.016, η^2^*_p_* = 0.311; IPL: *F*_(1,16)_ = 7.038, *p* = 0.017, η^2^*_p_* = 0.305: baseline: *F*_(1,16)_ = 9.313, *p* = 0.008, η^2^*_p_* = 0.368). In addition, there is a main effect of session within the angry condition (*F*_(2,32)_ = 8.298, *p* = 0.001, η^2^*_p_* = 0.342), which was driven by the difference between the IPL and baseline sessions (*p* = 0.006). Within the neutral condition, there is also a main effect of session (*F*_(2,32)_ = 6.824, *p* = 0.003, η^2^*_p_* = 0.299), which was driven by differences between both the PMv and IPL sessions (*p* = 0.046), as well as between the PMv and baseline sessions (*p* = 0.003).

### ROI analyses

#### Local effects of cTBS and emotion in rPMv and rIPL

A 2 × 3 repeated-measures ANOVA on the β values in rPMv showed neither a main effect of emotion (*F*_(1,16)_ = 0.603, *p* = 0.449, η^2^*_p_* = 0.036) nor a main effect of session (*F*_(2,32)_ = 0.902, *p* = 0.416, η^2^*_p_* = 0.053). There was, however, a significant emotion × session effect (*F*_(2,32)_ = 3.896, *p* = 0.031, η^2^*_p_* = 0.196). *Post hoc* tests to disentangle the interaction effect showed no main effects of emotion after PMv stimulation (*F*_(1,16)_ = 2.771, *p* = 0.115, η^2^*_p_* = 0.148), after IPL stimulation (*F*_(1,16)_ = 2.322, *p* = 0.147, η^2^*_p_* = 0.127), or during baseline (*F*_(1,16)_ = 2.783, *p* = 0.115, η^2^*_p_* = 0.148). Neither was there a main effect of session either in the angry condition (*F*_(1,16)_ = 2.376, *p* = 0.109, η^2^*_p_* = 0.129) or the neutral condition (*F*_(1,16)_ = 1.620, *p* = 0.214, η^2^*_p_* = 0.092).

In the ROI for rIPL, there was no main effect of emotion (*F*_(1,16)_ = 1.498, *p* = 0.239, η^2^*_p_* = 0.086), no main effect of session (*F*_(2,32)_ = 0.355, *p* = 0.704, η^2^*_p_* = 0.022), and no emotion × session interaction (*F*_(2,32)_ = 1.750, *p* = 0.190, η^2^*_p_* = 0.099).

#### Effect of cTBS and emotion in the amygdalae

A 2 × 3 repeated-measures ANOVA on the β values in left AMG (lAMG) showed a main effect of emotion (*F*_(1,16)_ = 19.561, *p* < 0.001, η^2^*_p_* = 0.550) as well as an emotion × session interaction effect (*F*_(2,32)_ = 3.922, *p* = 0.030, η^2^*_p_* = 0.197). There was no main effect of session (*F*_(2,32)_ = 0.497, *p* = 0.613, η^2^*_p_* = 0.030). *Post hoc* tests to disentangle the interaction revealed that while during both the PMv and the IPL sessions there was a significant difference between angry and neutral bodies (*F*_(1,16)_ = 13.533, *p* = 0.002, η^2^*_p_* = 0.458, and *F*_(1,16)_ = 19.085, *p* ≥ 0.001, η^2^*_p_* = 0.544, respectively), with stronger activation for angry than neutral bodies, this effect was absent during the baseline session (*F*_(1,16)_ = 0.066, *p* = 0.801, η^2^*_p_* = 0.004). In addition, pairwise comparison between sessions in the angry body condition showed that activation for angry bodies during the IPL session was significantly stronger than that during the baseline session (*p* = 0.033).

**Figure 5.**
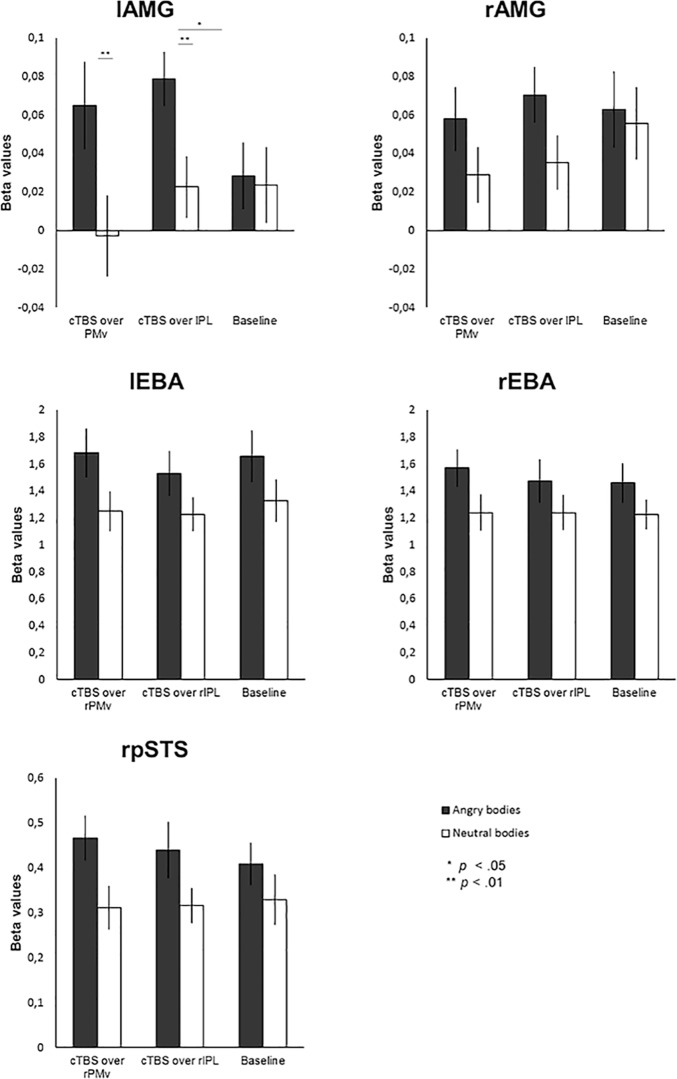
Results from ROI analysis based on individual anatomy (amygdala) or functional activity (EBA and pSTS). β values in rAMG, bilateral EBA, and rpSTS reflected a main effect of emotion, whereas in lAMG an interaction effect was also present. In lAMG, there was no effect of emotion at baseline, but this effect was evident for both cTBS sessions.

The analysis of the β values in right AMG (rAMG) showed a main effect of emotion (*F*_(1,16)_ = 6.67, *p* = 0.020, η^2^*_p_* = 0.294), with, on average, higher values for angry than neutral bodies (mean values, 0.064 and 0.040). There was neither a main effect of session (*F*_(2,32)_ = 0.440, *p* = 0.648, η^2^*_p_* = 0.027) nor an emotion × session interaction (*F*_(2,32)_ = 0.604, *p* = 0.553, η^2^*_p_* = 0.036; Fig. 5, results in AMG).

#### Effect of cTBS and emotion in bilateral extrastriate body area and right posterior superior temporal sulcus

A 2 × 3 repeated-measures ANOVA of the β values in right EBA revealed a main effect of emotion (*F*_(1,16)_ = 51.701, *p* < 0.001, η^2^*_p_* = 0.764), but no main effect of session (*F*_(2,32)_ = 0.295, *p* = 0.746, η^2^*_p_* = 0.018) or emotion × session interaction (*F*_(2,32)_ = 2.695, *p* = 0.083, η^2^*_p_* = 0.144). Results in left EBA again showed a main effect of emotion (*F*_(1,16)_ = 58.677, *p* < 0.001, η^2^*_p_* = 0.786), but not a main effect of session (*F*_(2,32)_ = 0.382, *p* = 0.685, η^2^*_p_* = 0.023) or an emotion × session interaction (*F*_(2,32)_ = 2.834, *p* = 0.074, η^2^*_p_* = 0.150).

Analysis of the β values in rpSTS revealed a main effect of emotion (*F*_(1,16)_ = 24.050, *p* < 0.001, η^2^*_p_* = 0.600), but no main effect of session (*F*_(2,32)_ = 0.141, *p* = 0.869, η^2^*_p_* = 0.009) or emotion × session interaction (*F*_(2,32)_ = 1.458, *p* = 0.248, η^2^*_p_* = 0.084; Fig. 4, all results of the ROI analysis).

## Discussion

We combined off-line TMS and fMRI to test the hypothesis that IPL and PMv are involved in processing of affective body expressions, and measured the possible interactions of these areas with AMG during the processing of such information. ROI analysis in lAMG revealed an interaction between the emotion portrayed in the stimuli and the stimulation site. Similar interactions were observed in clusters in SPL, PMv, SMA, cerebellum, and occipital cortex. Concerning lAMG, although there was no significant difference between angry and neutral bodies at baseline, this difference was significant after cTBS over both rPMv and rIPL. Functionally determined ROIs in EBA and pSTS showed only the main effects of emotion and did not interact with the stimulated regions, as their pattern of activation was unaffected by cTBS over either of the stimulation sites.

### Processing of affective body expressions in the amygdalae

Many classic studies have implicated the AMG as a region involved in the processing of threatening information (Ledoux, 2000), and more recently the importance of AMG interactions with cortical areas to respond effectively to threat has been suggested (Pessoa, 2017). Much of the research on AMG function has focused on the processing of faces and fear, but by now a broader picture of AMG function has emerged (Davis and Whalen, 2001). It appears that the AMG might also play a crucial role in the processing of emotional body stimuli (Hadjikhani and de Gelder, 2003, Hortensius et al., 2017), as previous work has shown that contrasting neutral and fearful body expressions show significant activation in AMG (Hadjikhani and de Gelder, 2003). In our baseline session, we did not find a significant difference between angry and neutral stimuli, in contrast with previous reports. However, current literature suggests that AMG activation in response to emotional body stimuli might depend on various task manipulations, such as task load and attention (de Gelder et al., 2012; Pessoa, 2017). For example, one study using an oddball paradigm and videos of actors opening a door either in a fearful or neutral manner (Grèzes et al., 2007) found only a general effect of seeing an action in the AMG, but no difference between fear and neutral images. Another experiment, which used the same stimuli as the current study, used an oddball paradigm comparing dynamic facial versus bodily expressions of emotions, and found that amygdala significantly distinguished between faces and bodies, but irrespective of the emotion that was expressed (Kret et al., 2011). Our findings for the baseline session are thus in line with previous work, as we compared passive viewing of dynamic neutral and angry bodies and found no significant differences in AMG between these two stimulus categories. Interestingly, our novel finding is that we found a significant difference between angry and neutral bodies after applying cTBS to either IPL or PMv, but not at the baseline session.

In one previous experiment, cTBS stimulation, limited to pSTS, demonstrated an effect on the BOLD signal in AMG, but only for a condition in which participants viewed dynamic facial stimuli (Pitcher et al., 2017). This particular study also explored the influence of stimuli depicting dynamic bodies, but, unlike our study, the stimuli consisted of only neutral movements. We find that the application of cTBS created a significantly stronger response to angry compared with neutral bodies, a result that fits well with previous findings of IPL stimulation. On-line TMS stimulation of IPL has been shown to result in increased, rather than decreased, sensitivity to emotional body stimuli (Engelen et al., 2015). Although speculative, it is possible that this previous finding might result from increased AMG sensitivity to emotional stimuli, as also observed in the current study. One possible explanation for these findings and the current results might be that at baseline IPL and PMv exert inhibition over AMG, and by means of TMS this inhibition is lifted. It is, for example, suggested that insufficient inhibition of AMG could lead to pathologic states, as this might lead to the expression of emotional responses in unwanted/unnecessary situations (Quirk and Gehlert, 2003). However, so far IPL and PMv have not been implicated in such an inhibition of AMG. With the current data, it is not possible to directly test this interpretation, but future studies comparing inhibitory and excitatory protocols over these areas, and examining subsequent effects on AMG, could clarify this.

The current findings support the proposed functional role of the AMG in interacting with motor-related regions in threatening situations and its relation to action readiness. So far, previous experiments have tried to provide evidence for such interactions by looking at (functional) connectivity between AMG and action-related regions. For example, by using diffusion tensor imaging, a study by Grèzes et al. (2014) revealed evidence for a pathway between AMG and several cortical motor areas, such a s pre-central gyrus and post-central gyrus motor structures and primary motor areas. Likewise, a study exploring psychophysical interactions between the AMG and other brain regions during the perception of emotional faces found increased functional coupling between AMG and premotor areas during perception of emotion faces versus neutral faces (Diano et al., 2017). Specifically, the perception of angry versus neutral faces increased functional coupling between AMG and IPL. These studies suggest that, under threatening circumstances, AMG has the option of communication with action-related areas through functional connectivity. Although exact circuits through which this might occur are to be established, the findings in the present study now suggest that, under conditions of perceived social threat, activity in the AMG can be altered by stimulation of either IPL or PMv.

### Accumulating evidence for parietal involvement in emotional body processing

Following on previous work establishing a causal role for IPL in emotion body processing (Engelen et al., 2015), we now show that IPL communicates with a number of regions, including AMG, to process the emotional content of actions. Considering its location and connections, IPL makes for a natural hub where emotion perception transitions into an action response. Several fMRI experiments observed emotion-specific activation within parietal cortex for both face stimuli (Grèzes et al., 2007; Kitada et al., 2010; Sarkheil et al., 2013) and body stimuli (Kana and Travers, 2012; Goldberg et al., 2014, 2015). Previously, an MEG experiment provided evidence for fast involvement (80-110 ms after stimulus onset) of IPL in discriminating between fearful and neutral body postures (Meeren et al., 2016). One fMRI study (Poyo Solanas et al., 2018) found significantly more activity in the IPL in response to body stimuli expressing fear in contrast to happiness, and moreover found increased responses in motor regions when fearful bodies were presented together with fearful faces, in contrast to bodies being presented in isolation. IPL activation was similarly found in a study contrasting the presentation of fearful bodies with two bodies expressing incongruent emotions (de Borst and de Gelder, 2016). Recent work by Mazzoni et al. (2017) demonstrated that anterior intraparietal sulcus holds a representation of affective body movements by using a state-dependent TMS paradigm. Other recent findings in IPL come from a study examining patients with Urbach–Wiethe disease (Hortensius et al., 2017). This patient group suffers from a lesion that is specific to basolateral AMG. In the experiment, the patient group was presented with face–body compounds that displayed either congruent or incongruent emotions between the face and the body. Irrespective of the emotion represented in the face, IPL showed increased activation if the body expressed fear. Together, a picture is emerging of the importance of IPL in emotional body processing, and results of the current study suggest that this may be implemented through its interactions with AMG.

### Recruitment of action observation and execution network for emotion body processing

Besides AMG, an interaction between stimulation site and valence of the stimulus was also observed in multiple clusters corresponding to areas known for their involvement in the observation and preparation of actions, including clusters in multiple parietal sites, SMA, and PM. SMA is an interesting area to consider within the emotional body-processing framework, as it has been suggested to act as interface between the limbic system and M1 in conveying emotional information. This was demonstrated in a TMS conditioning experiment in which larger motor-evoked potential amplitude was observed in an emotional visual condition if paired stimulation of SMA and M1 was used, compared with single pulses over M1 (Oliveri et al., 2003). In our data, we observed in SMA a significant difference between angry and neutral body stimuli at baseline. This difference was eliminated by IPL stimulation, and was reversed by the stimulation of PMv. A similar pattern was evident in the SPOC cluster showing a significant interaction effect. These findings show that these areas can be sensitive to emotional action information and that processing of this information can be altered by PMv and IPL stimulation. In this instance, they are affected by IPL and PMv differently since IPL stimulation seems to “block” or inhibit the emotion-related information in these areas while PMv stimulation seems to reverse it, although it is still unclear why this reversal would occur.

We additionally found several parietal clusters that showed an emotion-by-stimulation site interaction, including bilateral SPL, which is involved in both coordinating visuomotor actions and observation of actions (Culham and Valyear, 2006) and shows body-specific activation as well (Kret et al., 2011). Besides SPL, this interaction was also observed in SPOC and precuneus, of which the former codes for reachable space (Gallivan et al., 2009), and the latter is involved in tasks ranging from visuospatial imagery to the experience of agency (Cavanna and Trimble, 2006). The pattern of the interaction effect was slightly different in the SPL and in precuneus clusters compared with the SMA and SPOC cluster. Although they also showed a reversal for their response to angry and neutral body stimuli after PMv stimulation compared with baseline, the stimulation of IPL did not change activity to the stimuli compared with baseline.

### Threat-related modulation of the attention network

Although the role of both IPL and PMv in the observation and preparation of actions following perception of emotional signals is well established, it has to be noted that these two areas also belong to attentional networks. In terms of survival, having the ability to quickly and efficiently shift attention to relevant stimuli such as threatening body postures is highly beneficial. It has been proposed that, when reorientation toward threatening stimuli occurs, the ventral frontoparietal network has the ability to alter the activity within the dorsal frontoparietal network (Corbetta et al., 2008). The dorsal attention network is composed of right hemispheric parietal regions (IPS and SPL) and frontal regions around the frontal eye fields, whereas the ventral network comprises areas such as the temporoparietal junction, the ventral part of the supramarginal gyrus, and the ventral frontal cortex. The ventral attention network especially can be driven by the behavioral relevance of a stimulus, and IPL in particular has been linked to attention to emotion (Viviani, 2013). One TMS study investigating emotional spatial attention (Mulckhuyse et al., 2017) found that, when inhibitory stimulation was applied over posterior parietal cortex, reaction time costs to a threatening distractor were increased. Another fMRI study using emotional distractors found significant activation in IPL when emotional distractors of negative valence were presented. Furthermore, IPL activity was negatively correlated with activity in the AMG, suggesting that parietal cortex may exert control over AMG in situations in which distracting emotional material is present (Mitchell et al., 2008). Such an interpretation would fit the previously suggested interpretation of our findings in which IPL would exert inhibition over AMG. In our experimental design, the participants had no active task, and thus the threatening body postures may have been treated as “irrelevant,” leading to the suppression of AMG at baseline. cTBS over IPL could have lifted this inhibitory influence, resulting in the observed increased sensitivity of AMG to angry body postures.

Although the stimulation sites in this study were not selected as nodes of the attentional networks, they are indeed very close to or overlapping with the above-mentioned areas. This means that the current results might in part be explained by alterations of computations in the attentional networks. If, indeed, under normal circumstances areas such as IPL and PMv would be involved in the gating of irrelevant and the detection of relevant information, the “removal” of this filter could indeed cause greater sensitivity to emotion stimuli in the AMG. It is important to note that interpretations of the results in the light of either attention or action are not mutually exclusive, because in situations of threat such processes will likely go hand in hand and are difficult to disentangle. This also falls in line with the idea that areas, such as IPL and temporoparietal junction, might have overlapping functions such as attention and social cognition (Igelström and Graziano, 2017).

### Local and remote effects of cTBS

When investigating the local effects of cTBS stimulation, we found a significant emotion × session interaction in rPMv. However, neither rPMv nor rIPL showed a significant main effect for session. Interestingly, other studies have similarly not found clear decreased local activity following cTBS (Ott et al., 2011; van Nuenen et al., 2012). This absence of local effects is possibly caused by the fact that TMS effects can be highly variable between participants (Ridding and Ziemann, 2010) and might manifest in increased variance in the stimulated areas, rather than an overall suppression (Valchev et al., 2016).

In order to uncover any general remote effects of cTBS, we additionally explored the main effect of session in our whole-brain analysis. We found several clusters that displayed task-invariant changes in the BOLD signal, depending on the stimulation condition. Two clusters, namely vmPFC and PCC, showed a cTBS suppression of the BOLD signal compared with baseline irrespective of stimulation site. This suggests that cTBS in general resulted in a suppression of the default mode network (DMN), of which both vmPFC and PCC are parts (Buckner et al., 2008). The DMN generally shows greater activity at rest, and its suppression might result from stronger task engagement after cTBS. However, another important node of the DMN is the precuneus, which we also found to have a main effect of session, although suppression in this cluster was specific for IPL stimulation compared with both baseline and PMv stimulation. We also found that stimulation of rPMv suppresses activity in lSPL, whereas stimulation of rIPL leads to enhanced activity in lPMv. This finding seems to suggest interhemispheric interactions between premotor and parietal areas as a result of stimulation.

In the current experiment, we did not include sham cTBS stimulation in the baseline session, which might be seen as a limitation when it comes to the interpretation of stimulation-specific results. Including an appropriate control for active TMS stimulation is notoriously difficult, and there is no ideal solution for handling this issue (Duecker and Sack, 2015). Application of TMS is always accompanied by somatosensory effects and a clicking sound, and in some circumstances (depending on stimulation location and intensity) also peripheral nerve stimulation. Sham stimulation by means of a shielded sham coil provides a credible control for the clicking sound, but is easily distinguishable from active stimulation when it comes to somatosensation and peripheral nerve stimulation. However, when considering the importance of sham TMS, a distinction needs to be made between paradigms using either on-line or off-line stimulation. While effects induced by somatosensation and/or peripheral nerve stimulation associated with active stimulation could hinder the interpretation of results of on-line studies, this might be less so for off-line paradigms, as these effects are likely of a transient nature (e.g., short shifts in arousal or attention). Given that in the current experiment we only included participants that had previous experience with TMS, that sham cTBS in particular lacks some credibility compared with active stimulation (due to the lack of noticeable somatosensory effects and nerve stimulation that is otherwise present), and that our baseline session differed in duration from the other fMRI sessions due to the acquisition of the functional localizers, we opted to not include sham cTBS stimulation. Importantly, we chose to have two active stimulation sites in addition to the baseline session. In our results, we observe, both for the main effect of session as well as for the emotion × session interaction, clusters that have different responses for each of the stimulation sites compared with one another, as well as compared with sham. Nonetheless, we cannot rule out that awareness by the participants about lack of stimulation in the baseline session accounts for some of the effects observed in our results.

### Conclusions

In conclusion, by combining cTBS and fMRI, we were able to establish for the first time a causal relation between brain areas related to action and the perception of affective bodies. We showed specifically how, under conditions of perceived social threat, both IPL and PMv dynamically interact with AMG and with areas involved in observation and preparation of actions. IPL and PMv are likely candidates to play a key role in preparing fast adaptive action, as is often triggered by the sight of social threat.

## Synthesis

Reviewing Editor: Philippe Tobler, University of Zurich Decisions are customarily a result of the Reviewing Editor and the peer reviewers coming together and discussing their recommendations until a consensus is reached. When revisions are invited, a fact-based synthesis statement explaining their decision and outlining what is needed to prepare a revision will be listed below. The following reviewer(s) agreed to reveal their identity: Lennart Verhagen.

Apologies for the delay in getting back to you, which was partly due to illness and prior commitments of one of the reviewers. Although the reviewers appreciated the changes made to the version submitted to the Journal of Neuroscience (textual changes, conclusions closer to data, added clarifications), they both agreed that there were still serious issues with interpretation. These have to be addressed rigorously in case you decide to resubmit.

Engelen and colleagues present a combined neurostimulation-neuroimaging study of the neural circuitry supporting the processing of emotional whole-body dynamic stimuli. The results focus on two areas outside the conventional circuit for visual perception and emotion processing, right IPL and right PMv. The authors show that these regions are causally critical to modulate BOLD activation levels in the (left) amygdala, but not in the extrastriate body area or posterior superior temporal sulcus, in response to emotional threatening stimuli. After cTBS perturbation of either PMv or IPL the left amygdala was more strongly activated for angry over neutral dynamic bodily actions. The authors also report on whole-brain explorative analyses, revealing other areas that are sensitive to the cTBS perturbation and stimulus-valence interaction. Together, this study provides evidence for the modulation of a circuit involved in emotion perception by perceptuo-motor regions.

Major concerns:

1. Several issues revolve around the authors' interpretation of their findings.

a. Baseline activations. The amygdala shows no effect to emotional stimuli in the baseline condition, which conflicts with a large body of evidence. The authors should discuss possible reasons for this null effect. The finding that IPL and PMv TMS lead to emotion-related activation in the amygdala that is absent in the control condition seems to suggest that IPL and PMv (if undisrupted) suppress emotional responses in the amygdala. It is unclear why increased emotion-related activation in the amygdala should provide evidence for a role of IPS and PMv for translating emotion perception in to action, given the amygdala's role for emotion perception. If the IPL discriminates between fearful and neutral stimuli, as the authors state on p.19, then one would expect IPL TMS to reduce rather than increase emotion-related activation (see also major point 2). The authors do not do themselves a favor if they neglect these inconsistencies.

b. Sign of effects. The authors mention that they have added a paragraph in the discussion where they discuss the sign of the observed effects. Which paragraph might this have been? More importantly, one still wonders how the authors suggest to explain that cTBS over PMv-IPL leads to stronger (rather than weaker) activation in the left amygdala for angry movements. The authors hypothesize and conclude that PMv-IPL are involved in emotional body processing, but given that cTBS is generally regarded as a disruptive perturbation one could have expected that the amygdalae would be less prone to process emotional dynamic bodies. The opposite effect is observed. Why? Are the amygdalae engaged in a mutually inhibitory circuit with the sensorimotor network when at rest (or when no action should be executed)? On a related note, the authors link the deactivation in midline DMN nodes following cTBS to stronger task-engagement. Why would cTBS over PMv/IPL lead to stronger task engagement? Is this mediated through attentional mechanisms?

c. Connections between IPL/PMv and amygdala. The original review pointed out that the authors might have over-interpreted some contributions in the literature. Shortcomings remain in this regard. For example, they write: “Additionally, animal research shows connections between IPL and amygdala (AMG) (Clower, West, Lynch, & Strick, 2001)”. The cited paper is titled “The Inferior Parietal Lobule Is the Target of Output from the Superior Colliculus, Hippocampus, and Cerebellum” and does not reference the amygdala, let alone study its connections with the IPL (in fact, this study identifies second-order, rather than direct, projections). The authors also cite Toschi 2017. Again, this study does not report on amygdala-PMv connections, but on several networks, including one that includes the ventral pallidum. While the ventral pallidum is proximal to the extended amygdala nucleus, it is not part of the amygdala proper. In the discussion they also cite Roy et al 2009, but this study reveals a mixed finding, showing mostly negative or weakly positive functional coupling between amygdala and precentral gyrus. The reviewers are not aware of any study reporting on (direct) connections between the amygdala and the inferior parietal lobule or the ventral premotor cortex. Accordingly, phrases such as “Of particular interest [...] are parietal and premotor cortices, given their connectivity with the amygdala (AMG)” are either oversimplifying or misrepresenting current knowledge. The amygdalae may well play a critical role in adaptive (motor) behavior. Yet, given our current anatomical insights, this role is likely mediated through other circuits, such as the subcortical (basal ganglia) motor systems, rather than directly through posterior parietal or premotor cortices.

d. Labelling of IPL site. Both original reviewers highlighted that the IPL is a large and heterogenous region and asked for a better specification of the TMS site. The authors have added a few words, but have neglected to address the heterogeneity of the IPL, let alone to mention if they feel they stimulated over the angular gyrus or the supramarginal gyrus. Again, this is of importance because they have a differential role in motor preparation, attention, and the default mode network. Please also see minor point 1 below.

2. When arguing for their interpretation, the authors cite a study according to which the amygdala shows enhanced functional coupling with premotor areas during emotion processes (p.19). The authors should directly test whether TMS changed this connectivity by conducting PPI analyses with the stimulated IPL and PMv regions as seeds. It may not be necessary to report the results of this analysis in the manuscript if it does not reveal any interesting results, but these analyses might potentially improve our understanding of how exactly IPL and PMv stimulation modulates neural activation in the current study.

More minor concerns:

1. The authors rightfully point out that it is well established that the IPL and PMv circuits are strongly involved in motor processing. However, both original reviewers pointed out that these circuits are also involved in perceptual and attentional processing. The authors highlight that a similar passive viewing task has resulted in IPL/PMv activation in an earlier study. That is indeed highly indicative, but it does not exclude the possibility that the attentional and perceptual computations of these regions played a role here. In fact, given the subjects' requirement not to act, it would not come as a surprise if the IPL-PMv contributions where mostly limited to action perception computations. Or, alternatively, if the cTBS stimulation led to a change in attentional efficacy, thereby affecting processing.

2. The authors rightly mention on several occasions the role of IPL in both action observation and action execution in tandem. The reviewers are not arguing that the authors should re-do the study with a proper motor task. However, they encourage the authors to take attentional and perceptual (action observation) computations more seriously in the interpretation of the results. On a related note, the title still refers to “motor preparation networks”, but the abstract refers in a more balanced fashion to “areas involved in action perception and preparation”.

3. As subjects were just passively viewing the stimuli, how can the authors be sure that subjects actually attended to the stimuli?

4. Was the order of the angry and neutral blocks randomised within a run?

5. Please specify in more detail the task active regressors of the GLM. Were these mini-block regressors covering the 6 TRs of the stimuli (but excluding the 6 TRs with fixation)?

6. Results, main effect of emotion. Does the “occipital cluster in the right hemisphere (cluster 5 in table 2)” refer to SPOC (the fifth cluster in table 2) or to the ‘widespread occipital’ cluster (the first cluster in table 2)?

7. Given that all subjects participated in three imaging sessions and given that amygdala activation to emotional stimuli has been reported to adapt rapidly (e.g., Breiter et al., 1996, Neuron; LaBar et al., 1998, Neuron), the authors may want to test for task repetition/habituation effects.

8. The functional localizer tasks for EBA and rpSTS come out of the blue. If the authors had apriori hypotheses regarding how TMS should affect activation in EBA and rpSTS, these predictions should be reported.
